# Knowledge and Will: An Explorative Study on the Implementation of School-Wide Positive Behavior Support in Sweden

**DOI:** 10.3389/fpsyg.2021.618099

**Published:** 2021-02-16

**Authors:** Kata Nylén, Martin Karlberg, Nina Klang, Terje Ogden

**Affiliations:** ^1^Magelungen Utveckling, Stockholm, Sweden; ^2^Department of Education, Uppsala University, Uppsala, Sweden; ^3^Norwegian Center for Child Behavioral Development, Oslo, Norway

**Keywords:** TDF, PALS, IBIS, COM-B, SWPBIS, school implementation, program adaptation, PBIS

## Abstract

School-wide positive behavior support (SWPBIS) is a well-evaluated school approach to promoting a positive school climate and decreasing problem behaviors. Initial implementation is one of the most critical stages of program implementation. In this qualitative study, the initial implementation of SWPBIS in Swedish schools was studied using an implementation model of behavior change as guidance for interviews and analyses. The study makes significant contributions to previous research as little is known of the implementation of SWPBIS in Swedish context. Focus-group interviews were conducted with 59 professionals on implementation teams from nine schools. Themes were extracted according to implementation team members' perceptions and descriptions of how the initial implementation was carried out. The results of this study revealed relevant themes within the three domains of Capability, Opportunity, and Motivation. Core features were found under the themes of knowledge and experience of similar evidence-based programs, process or result orientation, time, manual content, organizational prerequisites, team functioning, implementation leadership, program as a unifying factor, program aligning with staff beliefs, plausible expectations, and emotional reinforcement. Results are discussed in terms of how they can be used in continuing to develop the Swedish model of SWPBIS. Implications regarding implementation in Swedish schools are discussed, as is the applicability of the model of behavior change for studying implementation in schools.

## Introduction

Positive Behavior Intervention and Support (PBIS) is a systems-level multi-tiered approach that encourages socially acceptable student behaviors by creating a safe and supportive social climate (Horner et al., 2017). The evidence base of the PBIS is extensive, and is related to positive behavioral, academic, and organizational outcomes (Bradshaw et al., 2010, 2012; Horner et al., 2010; Solomon et al., 2012; Gage et al., 2018; Mitchell et al., 2018). However, the effectiveness of PBIS is dependent on the quality of its implementation (Bradshaw et al., 2008; Horner et al., 2010; Dix et al., 2012; Flannery et al., 2014; McIntosh et al., 2014; Sørlie et al., 2018). In fact, schools have been shown to vary widely in their fidelity to and implementation rates of the approach (Buzhardt et al., 2006; Lee and Gage, 2020), and there is often a gap between research and practice when preventive programs are implemented in schools (Wandersman et al., 2008). Schools are at the greatest risk of abandoning PBIS within the first 2 years of implementation (Nese et al., 2016). The aim of this study was to add to the empirical literature on the initial implementation of PBIS in Swedish schools by exploring what core features in the implementation teams perceive as hindering or enabling in the initial implementation of the Swedish PBIS model. PBIS consist of three tiers and the Swedish schools had only started implementing the first universal tier while the study was conducted. Consequently, this study explores the initial adaption of the universal tier of PBIS. Using the Theoretical Domains Framework (TDF) and the Capability, Opportunity, Motivation - Behavior (COM-B) model (Michie et al., 2011) as guidance, the study also explores the usefulness of the COM-B model and the TDF to enhance the understanding of the initial implementation of School-wide PBIS (SWPBIS) in a Swedish context. TDF, a comprehensive framework based on psychological knowledge of behavior change, was chosen as a new framework of exploration, as it gives the possibility to enhance the understanding of implementation of SWPBS on a theoretical and behavioral level (Atkins et al., 2017). Lack of fidelity and sustainability is common scenarios in implementation in schools, especially in imported innovations (Ingemarson et al., 2014; Bodin et al., 2016). This exploratory study contributes to the literature with a greater understanding of initial implementation, cultural adaption of SWPBS in a Swedish context and the usefulness of TDF and COM – B in studying implementation of evidence based practices in schools.

## Background

School-Wide Positive Behavioral Interventions and Supports (SWPBIS) is an approach to addressing problem behaviors and promoting social behaviors by targeting school and classroom organization (Horner et al., 2017). SWPBIS refers to Tier 1, with interventions directed at all students and all school personnel (Noltemeyer et al., 2018). In this tier, positive expectations, rules, and classroom management strategies are introduced to prevent challenging behaviors and encourage prosocial behaviors. After Tier 1 has been implemented, students in need of additional support receive this in accordance with a continuum of support in Tier 2, providing group interventions to students with similar needs, and Tier 3, supporting individual students through individualized interventions. PBIS is not a curriculum, intervention, or manualized program but rather a framework for organizing prosocial behavioral support that results in social, behavioral, and academic success for all students at a school (Sugai and Horner, 2020). Key elements in the framework include (a) using data, as academic and behavioral indicators, to guide decision-making; (b) applying established evidence from the behavioral and biomedical sciences to address problem behaviors; (c) selecting research-validated practices based on data on student behavior; and (d) using a systems change approach whereby whole school routines and resources are accounted for (Sugai et al., 2009). The implementation of PBIS is guided through well-defined implementation stages, from an initial exploration of the school readiness for the intervention to successively scaling up the intervention (Sugai and Horner, 2020). Prior to implementation start, schools are required to conduct self-assessment to determine need gaps within the organization and develop an implementation plan (Sugai et al., 2009). The school leadership teams are responsible for ensuring that the new interventions fit with the school's organizational context, if necessary developing organizational structures for implementation and allocating time and resources for successful implementation. The implementation process is to be followed up through continuous data collection. The implementation is team-based, and an implementation team, formed at each school, informs and coaches the school personnel in the key components. Thus, the team is the core element of the school implementation. The school team receives guidance from a certified coach. Before upscaling the intervention, it is necessary to establish the training capacity by recruiting external trainers to assist local school teams, the coaching capacity by tailoring and adapting the content to fit local needs, and the evaluation capacity by continuously assessing the fidelity of the implementation (Horner et al., 2014).

While the research base concerning SWPBIS in the United States (US) is extensive, fewer studies have been published in Europe (Sørlie and Ogden, 2015; Närhi et al., 2017; Wienen et al., 2019). However, these studies have shown promising results concerning student behavior, classroom climate, peer relations, and teacher behaviors. This study, the first on the implementation of the SWPBIS framework in Sweden, was based on the Norwegian manualized program N-PALS, based on the SWPBIS framework. The Norwegian model shares its core components with the original SWPBIS framework (Sørlie and Ogden, 2015). The model combines preventive intervention strategies for all students at Tier 1 with intensive and individualized strategies for students who need more support at Tiers 2 and 3. The implementation process is developmental in nature and schools need to have implemented Tier 1 before starting to implement Tier 2 and 3. At the universal level (Tier 1), the program's core components constitute: (a) positive behavior support strategies, including positive expectations and classroom rules, which are followed up with positive feedback and encouragement; (b) a system for monitoring student behavior; (c) school-wide corrections using consequences; (d) instruction in classroom management skills for teachers; and (e) strategies for collaboration with parents. In this study, the initial adaption of the universal tier of the N-PALS program was studied. Similar to the original SWPBIS, the N-PALS has a well-established implementation plan. Each school's readiness for implementation is to be assessed, and consent from at least 80% of the school staff is required prior to implementation initiatives (Norwegian Center for Child Behavioral Development, 2017).

The Swedish IBIS (Inclusive Behavioral Support in Schools) program manual is largely a translation of N-PALS. The differences between the Norwegian and Swedish versions are three-fold: (a) references to the Norwegian Education Act and Norwegian policy documents for the school have been replaced with their Swedish counterparts, (b) the Swedish version has fewer illustrations and a less advanced layout, and (c) IBIS aims and goals are primarily intended to support *inclusive* behaviors, something that is considered to be of the greatest importance in Sweden.

Swedish coaches responsible for the implementation of the program received training at the Norwegian Center for Child Behavioral Development (NCCBD). Although Sweden and Norway have similar characteristics in terms of the conditions of compulsory school education and the teaching profession, Sweden may constitute a specific context for the implementation of the SWPBIS framework. In the Swedish school system, teachers are afforded a considerable degree of independence in regard to teaching methods (Helgøy and Homme, 2007). There are some clear characteristics of the Swedish school: requirement of scientific base, inclusion, and the system of independent schools. In recent decades, the evidence movement has grown in Sweden (Adolfsson and Sundberg, 2018). The Education Act states that all activities in the school must be based on science. Accordingly, many school leaders request evidence when planning to implement a new program or approach. At the same time, there is some opposition to the evidence movement. Some believe that the ongoing evidence movement takes place from a top-down perspective and that it reduces teachers' independence and poses a threat to their expertise and profession. Critics of the evidence movement believe, for example, that it can be difficult to generalize the results of a study at one group of schools to other schools (Hammersley, 2007). A common objection, which is of course relevant, is that programs that have been shown to be effective in the USA do not have to work in the same way in Sweden. Another objection is that it can be difficult to be sure about the evidence for a program that has been adapted to local conditions. Critics fear that important program components can get lost in the adaptation process.

The Swedish school system is characterized by a strong effort to include students who for various reasons need support for their learning and development. According to Chapter 3 of the Swedish Education Act, students are entitled to support within the framework of regular teaching. School politicians, school leaders and teachers argue for increased inclusion, but many of those who are practically responsible for the inclusion lack effective methods, programs, and approaches. This is one reason why there can be a strong support for inclusion and, at the same time, a huge number of excluded students. Schools and teachers are unable to adapt instruction in accordance to students' differences.

Sweden has a very liberal school market, where practically anyone can start a school. This is reflected in the fact that there are a large number of small idea-driven schools but also a number of large school organizations. A government investigation (SOU, 2020:28) showed that equality in Swedish schools is decreasing and that segregation is increasing. Among other things, this is shown in the fact that low-performing students and students in need of behavioral support attend municipal schools, at the same time as high-performing students without the need for extra adaptation and special support attend private schools. Many teachers in the municipal schools experience a stressful work situation with many students in need of support. At the same time, the teacher to student ratio in the private schools is lower, which affects the teachers' ability to teach and manage the classroom. Regardless of whether the teachers work in a municipal or a private school, there is a great need among the teachers to develop their skills in working with students in need of special support and to handle behavior concerns (Karlberg and Bezzina, 2020).

At the same time, children's mental health and school violence constitute major issues in Swedish schools (Galanti et al., 2016). Previous studies have also shown that Swedish schools trying to implement evidence-based practice have had difficulty succeeding (Ingemarson et al., 2014; Bodin et al., 2016). It is therefore interesting to explore how Swedish school professionals perceive the initial implementation of an intervention framework like SWPBIS.

### Implementation of SWPBIS in Schools

Implementation science, the study of how practice is used, adopted, and sustained, has been identified as the most important research challenge within educational science for the next decades (McIntosh et al., 2013). While the gap between research and practice is not unique to schools, they do constitute a specific environment due to a diverse population of students to be served and competing agendas in the choice of evidence-based practice (Wandersman et al., 2008; Cook and Odom, 2013; Forman et al., 2013). It takes time to implement evidence-based programs and several stages in implementation have been identified in a process largely used in the research on SWPBS implementation (Fixsen et al., 2011). During the first stage, the exploration phase, problems in local contexts are identified, as are potential solutions to these problems, in the form of evidence-based practice. During the second stage, the installation phase, potential evidence-based programs are explored and resources for implementation are secured. It is not until the third stage, the initial implementation stage, that resources and leadership are redirected to the implementation of a new program and staff competencies in the new program are developed. This is the most turbulent time, as the program's content and associated routines are new to all participants. It is also at this stage that there is a risk that an evidence-based program will be abandoned (Fixsen et al., 2013). At this point, the intervention implementors may need to go back to the exploration and installation phases. If the initial implementation is successful, the organization enters a full implementation stage when half or more of the professionals meet the intervention program's professional standards.

A core issue in implementation is a fit between the local context and a program innovation. Moore et al. (2013) identify the need for *philosophical fit* (the match between staff beliefs and the value base of the program) as well as *practical fit* (whether the organization has the necessary time, resources, and practical solutions). For there to be a change to the system, there is a need for regular feedback from local implementation teams to researchers and policy-makers concerning what is hindering the process of implementing an evidence-based program (Fixsen et al., 2013). This fit between the local context and the program is central in the discussion of program fidelity and program adaptation. According to von Thiele Schwarz et al. (2019), on the one hand programs need to be implemented with fidelity to be enable to draw valid conclusions specific to the mechanisms of change inherent in core components of the program. On the other hand, adaptations in programs are vital for ensuring intervention-context fit and thus value to the recipients of an intervention. In fact, previous research states that schools appear to be more likely to sustain implementation when they tailor the PBIS practice to fit the school culture and adapt its content to the specific context and needs (Flannery et al., 2009; McIntosh et al., 2013; Horner et al., 2014; Pas et al., 2015). Thus, in this regard, it is important to explore how the school professionals view the SWPBIS at the initial stage of implementation and what adaptations may be needed in applying it in the Swedish school context.

### Enablers and Barriers in Initial Implementation of SWPBS

Despite evidence suggesting that SWPBS improves behavior and academic achievement in students of all ages and is associated with positive long-term outcomes, many schools still struggle with challenges in the implementation process (Jarboe, 2020). Previous research has therefore tried to identify barriers to and enablers of successful implementation and sustainability. These studies have found core features, enabling as well as hindering, that are integral to initial and sustained implementation over time. In the implementation of SWPBS, both *individual factors* and *organizational factors* have been identified.

Individual factors are staff being comfortable starting interventions on their own (Chitiyo and Wheeler, 2009; Lohrmann et al., 2013; Tyre and Feuerborn, 2017), sufficient knowledge (Kincaid et al., 2007; Chitiyo and Wheeler, 2009; Bambara et al., 2012; Lohrmann et al., 2013; Horner et al., 2014; Pas et al., 2015; George et al., 2018) and staff buy-in regarding a commitment to the principles behind the philosophy of the intervention (Forman et al., 2009).

Organizational factors are resources like time and money (Kincaid et al., 2007; Lohrmann et al., 2008; Chitiyo and Wheeler, 2009; Flannery et al., 2009; McIntosh et al., 2013; Horner et al., 2014; Pas et al., 2015; Tyre and Feuerborn, 2017; Goodman-Scott et al., 2018; Tyre et al., 2018), proper planning of staff and resources and proper training (Kincaid et al., 2007; Bambara et al., 2012; McIntosh et al., 2013; Pas et al., 2015), leadership, administrative and technical support, and logistical factors (Kincaid et al., 2007; Lohrmann et al., 2008; Chitiyo and Wheeler, 2009; Bambara et al., 2012; Tyre and Feuerborn, 2017; George et al., 2018; Goodman-Scott et al., 2018; Tyre et al., 2018). Previous studies on enablers and barriers in the implementation of SWPBS state that practical fit and organizational factors such as leadership, administrative supports, and logistical factors are important and act as recurring barriers (Kincaid et al., 2007; Chitiyo and Wheeler, 2009; Fallon et al., 2014; George et al., 2018; Tyre et al., 2018). This is strengthened by research on the implementation of evidence-based practice in schools in general (Greenhalgh et al., 2004; Langley et al., 2010). Therefore, many studies highlight the importance of ensuring that staff buy in, technical support and proper team training are established early. These studies have shown the importance of assessing how the school staff perceives PBIS practices as an important aspect of implementation, especially during the initial implementation period (Kincaid et al., 2007; Lohrmann et al., 2008; Flannery et al., 2009; Bambara et al., 2012; McIntosh et al., 2013; Pas et al., 2015).

In sum, previous research has shown the importance of organizational prerequisites. At the same time, organizational factors are often described as barriers and can be difficult to change in the initial implementation phase. Therefore, more information is needed on how school implementation teams can facilitate staff behavior change in line with the implementation of SWPBIS, despite the organizational challenges.

### A Behavioral Approach to Implementation

Implementation involves behavior change, and the implementation of SWPBS must primarily result in school staff behavior change. Thus, many factors need to be in place and collaborating with each other for the behavior change to occur (Francis et al., 2012; French et al., 2012). The Theoretical Domains Framework (TDF) is an integrative implementation framework, developed to understand behaviors so that implementation processes can be effectively targeted for change (Michie et al., 2011). The TDF offers a theoretical lens through which to understand and investigate influences on behavior in the context in which they occur. Based on psychological knowledge of behavior change, it offers a unique framework for exploring the barriers to and enablers of implementation. The theoretical base makes the framework relevant for investigating implementation problems and informing implementation interventions (Atkins et al., 2017).

**Figure 1:**
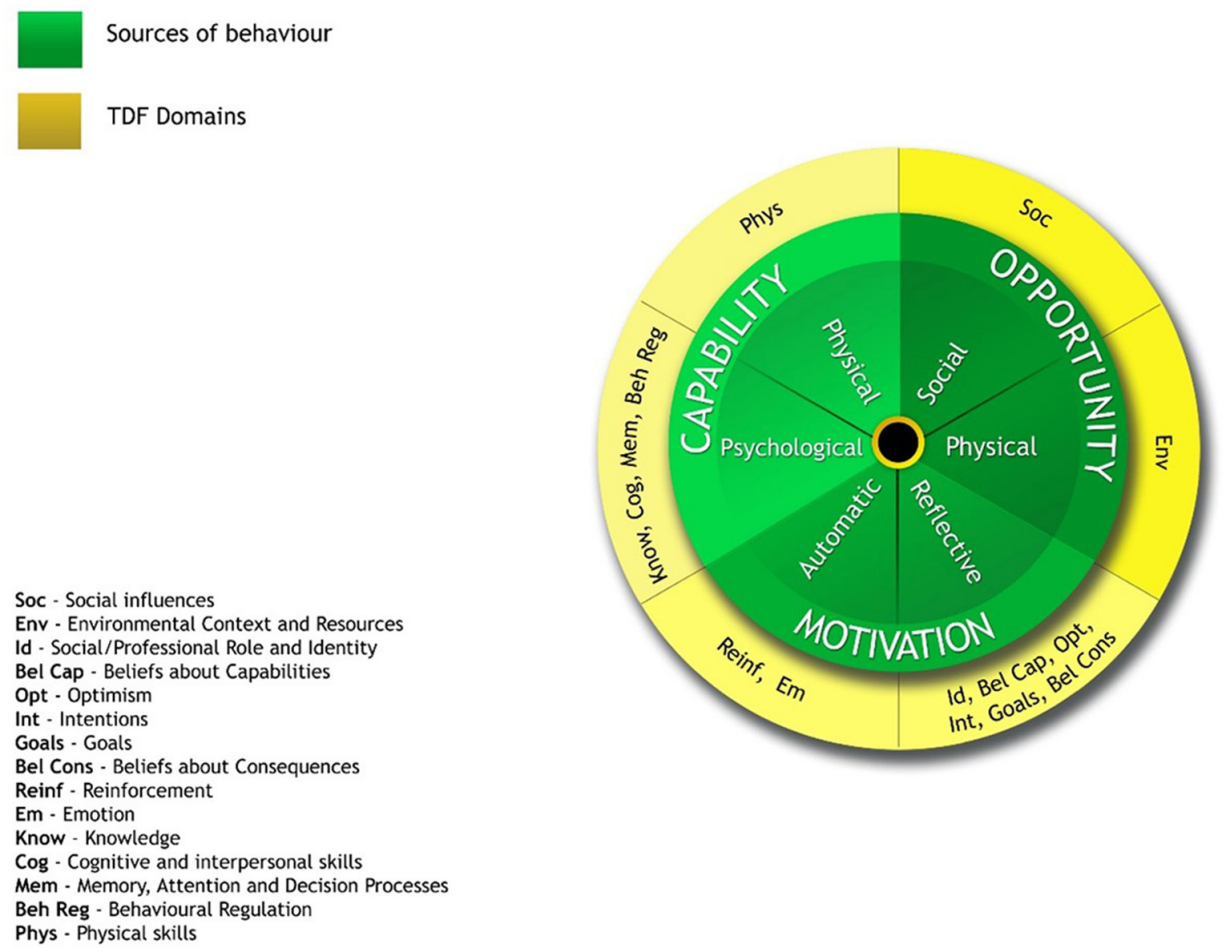
TDF Theoretical Domains mapped onto the COM-B model of behavior change (Michie et al., 2014).

The TDF disentangles 33 psychological and organizational theories and 128 constructs that may explain behavior change into 14 domains supported by psychological theory (Michie et al., 2005; Cane et al., 2012). The 14 domains of the TDF can be distilled into three core components: Capability (C), Opportunity (O), and Motivation (M). These components form the COM-B model, an implementation model based on the concept that behavior (B) change results from the interaction between the physical and psychological capability (C) needed to perform the behavior, the physical and social environmental opportunities (O) needed to undertake the behavior, and the external and intrinsic motivation (M) needed to utilize the opportunities and capabilities (Michie et al., 2011). Figure 1 shows how the 14 TDF domains are distributed over the COM-B areas. The Capability component, which includes both physical and psychological capability, encompasses the domains of knowledge, memory, attention and decision process, cognitive and interpersonal skills, behavioral regulation, and physical skills. The Opportunity component includes social and physical opportunities, and encompasses the domains of environmental context and resources and social influences. The Motivation component includes both reflective and automatic motivation, and encompasses the domains of social/professional role and identity, beliefs about capabilities, optimism, beliefs about consequences, reinforcement, emotion, intentions, and goals (Michie et al., 2014).

While all these components are interdependent, in combination they can provide an understanding of why a target behavior is not performed as desired and offer help in identifying which components should be addressed in order to change a behavior, or support the maintenance of an adopted behavior (Francis et al., 2012; French et al., 2012). The TDF was originally developed to identify influences on health professional clinical behavior change (Francis et al., 2012), but has recently been applied in many areas other than the health care sector (Davis et al., 2015; Gainforth et al., 2016) as well as in the school context (e.g., Clarke et al., 2015; Bayley et al., 2017; Martin and Murtagh, 2017; Hatfield et al., 2019). However, the TDF has not yet been used to explore the initial implementation of SWPBS. TDF and COM – B has largely been applied at explorative qualitative studies to elicit professionals' perceptions of TDF related barriers and enablers and is an appropriate framework to use in when little is known about an implementation problem (Atkins et al., 2017). It is a well-cited and used framework (Atkins et al., 2017) and given its contributions to understand, design and explore implementation in other contexts and of other interventions, it is interesting to investigate its usefulness in exploring implementation of SWPBS.

As shown above, several barriers to and enablers of the implementation of SWPBS in schools have been identified in previous research. More common frameworks used to investigate the implementation of SWPBIS, for example Fixsen et al. (2013), have made major contributions to the field in understanding core organizational features hindering and enabling the implementation process. At the same time organizational factors are often difficult to change in the initial phase of implementation and little is known about how implementation teams can compensate for these barriers and promote the enablers in an initial implementation process. Using a new framework in exploration, this study adds to the understanding of initial implementation and cultural adaption. No study, to the best of our knowledge, has yet examined the initial implementation of SWPBS in a Swedish context or used the TDF as a framework for investigation. Using the COM-B model and the TDF, this study examines the perceptions of implementation teams at nine Swedish schools in the initial implementation of SWPBS, in order to add to the empirical literature on core features in this context as well as the usefulness of the TDF and COM-B model in exploring SWPBIS implementation.

## Purpose of the Study

The aim of the study was to explore school implementation teams' perceptions of core factors in the initial implementation of SWPBIS at nine Swedish schools. In order to gain a greater understanding of factors that hinder or enable the initial implementation processes and cultural adaption of evidence based practices in schools two research questions were explored:

What core features do the implementation teams perceive as hindering or enabling in the initial implementation of the Swedish version of SWPBS?

How can the COM-B model and the TDF framework enhance the understanding of the initial implementation of SWPBS in a Swedish context?

## Method

Semi-structured interviews were conducted with nine school teams, while they were in the initial implementation of the Swedish model of SWPBIS, called IBIS.

### Context of the Study

The interviews were held in an initial phase of implementation. Below follows a description of the process that the participants had been through before meeting the researchers.

#### Exploration and Choice of Intervention

The preparations for implementation in the schools were extensive, and lasted about 3 months. Representatives of the municipality contacted Uppsala University to investigate the possibility to develop and implement an intervention aimed at creating a good learning environment for students and a good working environment for teachers. According to the municipality's representatives, there was an extensive need for the support and development of several schools' work with classroom management and inclusive practice. After searching for an effective and practical solution and studying several prevention programs, the choice fell on PBIS. The framework was in good agreement with the municipality's wishes, and the fact that it had already been successfully implemented in Norway cemented the decision. At a number of meetings between representatives of Uppsala municipality and researchers from Uppsala University, recruitment, the dissemination of information, and implementation were planned.

#### Installation and Inclusion Criteria

**Figure 2:**
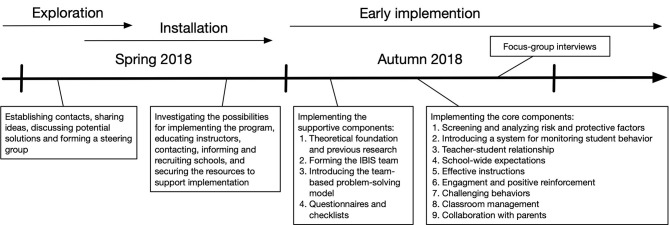
Implementation procedure of SWPBIS in the Swedish context.

Partly in parallel with the exploration phase, the installation phase began in a step-by-step process; see Figure 2. First, information was sent to all municipal and private schools in the municipality. Subsequently, interested principals were invited to an information meeting. At the first of several meetings, all principals in the municipality were briefly informed about IBIS and the plans to implement it. They were also informed about the inclusion criteria for the study, in accordance with Arnesen et al. (2003):

- Schools had to experience a need to develop positive behaviors and promote a supportive learning environment.
- At least 80% of school staff had to be positive to participation in implementing the program.
- School management had to support the implementation and actively participate in it.
- The schools needed to identify and define at least one target for improvement at the school.
- Parents, school administration, and other professional groups had to support the implementation and actively participate in it.
- The school had to be willing to allocate enough time and resources in order to be able to focus on implementing the program over the course of 3 years.
- The school had to be willing to contribute to data collection for evaluation.

All the participating schools agreed on the inclusion criteria. In the next step, interested principals were summoned to an information meeting where they were given detailed information about the supportive components in IBIS, in accordance with Arnesen et al. (2003); i.e., (a) the rationale and the theoretical foundation for the program, (b) the plan for implementation, and (c) a summary of previous research on SWPBIS and N-PALS. The principals were informed that the implementation of the IBIS program would take at least 3 years. Principals who were still interested were given the task of informing their school staff about IBIS and investigating the staff's attitudes regarding implementation of IBIS. Schools that were interested were then given the task of creating local teams at the school (IBIS teams), which would be responsible for the implementation of IBIS at the school. The IBIS team would consist of five to six people, including the principal and representatives of different occupational categories and already existing teams, as well as personnel working on student health. During the first year, nine schools registered for the IBIS project. In preparation for the IBIS implementation, the schools' IBIS team received an introductory lecture concerning the supportive components of the program (Arnesen et al., 2003). On this occasion, the schools were also paired with their future instructors. As a final step in the preparation for implementing the IBIS program, all personnel at the nine schools attended a lecture with the same content the principal and the IBIS teams had received. This was done a couple of weeks before the 2018 summer holidays. The plan was that all school personnel should have a good idea of the program's foundation and an idea of how the implementation would proceed. Among other things, all school staff were informed that the implementation of IBIS would take at least 3–4 years and that it could take a while before they started seeing visible effects of the program. After this lecture, there were great expectations among the school personnel as to what the implementation of IBIS would result in.

At the same time as the schools were being prepared for implementation, three instructors who would be responsible for the training and supervision of the schools' IBIS team were trained. The instructors were trained for a total of 8 days at the Norwegian Center for Child Behavioral Development in Oslo (NCCBD), the organization responsible for training instructors and implementing SWPBIS in Norway. After the training, during the implementation, the instructors had supervisors who had been trained at NCCBD.

#### Initial Implementation Procedures

As seen in Figure 2, during the two first months of initial implementation the work was devoted to implementing the supportive components, in accordance with Arnesen et al. (2003): (a) forming the IBIS team, (b) introducing the team-based problem-solving model, (c) investigating the risk and protective factors of the schools, and (d) filling in checklists and conducting surveys. During this period only the IBIS teams, not the school staff or students, were involved in the implementation.

The first part of the implementation saw the IBIS teams meeting their instructors and gaining access to the manual. The instructions to the instructors were to meet the school teams 1–2 times a month during a total of 3 h. With the support of the manual, the IBIS teams were informed about the program's theoretical foundation and research support. Among other things, the teams discussed the causes of and interventions for internalizing and externalizing behaviors, the importance of working proactively, and how the teams could use the Response-to-Intervention model in their preventive work. Great emphasis was placed on providing information to the teams on how to implement the program.

In the next step, the IBIS teams worked to define their area of responsibility and determine their roles in the team. The team also developed a plan for how they would interact with the school's other staff, how they would use the school's resources, and how they would coordinate the IBIS program with the school's other teams and assignments. Finally, the IBIS team was taught how to use the team-based solution model for their work.

In the third stage, the IBIS teams began to use a system for monitoring student behavior. They started using various tools for screening and evaluation, such as forms for mapping risk and protective factors in the school and checklists to evaluate implementation and planning.

In the fourth stage, some of the program's core components were addressed: teacher-student relationship, school-wide expectations, effective instructions, engagement, and the reinforcement of student behaviors. Great focus was placed on creating good relationships between teachers and students. Furthermore, the schools worked extensively to create and anchor rules in the schools, and to formulate these rules in the form of expectations. In this step, the students took part in systematic training in social skills. Furthermore, the teachers worked on developing effective and clear instructions and prompts. The final part of this step consisted of all school staff working systematically to increase their involvement in the students' and the school's activities and in providing reinforcement when students behaved in accordance with the school's rules and expectations.

In the fifth step, another of the program's core components was addressed: challenging behaviors. This step contained, for example, various examples of how the school can work to enforce consequences for challenging behaviors. Great focus was placed on teachers physically intervening as little as possible, developing teachers' abilities to prevent challenging behaviors by working systematically with classroom routines and strategies, and preventing future behavioral problems by analyzing antecedents and consequences of students' challenging behaviors.

In the sixth stage, classroom management was addressed. Here, the instructors and the IBIS team worked to develop teachers' classroom management skills, including organizing classrooms, clarifying routines, getting students to pay attention, and making use of predictable responses.

In the last step, strategies for collaboration between the school and parents were implemented. Among other things, the IBIS teams and school staff worked to clarify expectations for both school and guardians. They also worked on creating a system of communication between parents and the school.

Table 1 presents the number of schools that had successfully implemented the various components of the program, as rated by the IBIS instructors.

**Table 1:** Overview of the number of schools that successfully managed to implement the supporting and core components.

**Supporting components according to Arnesen et al. (2003)**	**Core components according to Sørlie and Ogden (2015)**	**Chapter in the manual**	**Number of schools successfully implementing the component**
Theoretical foundation		Rationale and schedule	9
		Theoretical foundation	9
Research base		Previous research	4
Establishing a behavioral support team		Team-based organization	9
	A system for monitoring student behavior	Tools for screening and evaluation	9
	Positive behavior support strategies, including positive expectations and classroom rules, which are followed up with positive feedback and encouragement	Core components: Teacher-Student Relationship	9
		Core components: School-wide expectations	9
		Core components: Effective instructions	9
		Core components: Reinforcement and engagement	9
	School-wide corrections using consequences	Core components: Challenging behaviors	2
	Instruction in classroom management skills for teachers	Classroom management	3
	Strategies for collaboration with parents	Home and school collaboration	1

*The data in the table are based on the instructors' ratings*.

### Participants

A total of 59 IBIS team members from nine different schools in the Swedish municipality of Uppsala participated in the study. Participants were recruited as the first schools piloting the IBIS program. Team members from all schools participated in the study. No participants refused to participate.

Team members were recruited by the school principal, and the different professions/roles represented were: teacher, school counselor, special education teacher, leisure educator, school counselor, and student health providers (e.g., school psychologist or school nurse). Each team included the school's principal. Detailed information about the schools can be seen in Table 2.

**Table 2:** Detailed information about the schools.

	**Number of pupils**	**Level**	**Parents with post-secondary education (%)**	**Students meeting the grade requirements^a^ (%)**	**Students with foreign background^b^ (%)**	**Students per teacher**	**Licensed teachers (%)**	**Length of interview**
School 1	376	Secondary	47	98.7	9	14.2	94.1	39:36
School 2	110	Primary	53	93.9	56	11.0	74.0	37:36
School 3	515	Secondary	57	92.3	23	16.9	93.4	38:49
School 4	391	Primary	70	96,2	13	18.5	85.8	28:34
School 5	242	Primary	32	78.7	79	18.3	86.0	39:10
School 6	192	Primary	88	93.8	28	14.5	90.9	40:57
School 7	279	Primary	74	94,5	8	16.2	100	56:24
School 8	353	Primary	59	90,5	26	14.4	96.3	36:40
School 9	615	Secondary	61	90,5	14	14.3	84.2	32:12
Average in the municipality	23,574 in total	Primary and Secondary	47	94,3	26	13.9	85.4	

*Data from the National Agency for Education (www.skolverket.se/skolutveckling/statistik, retrieved December 21, 2020)*.

a *Students meeting the grade requirements at the national tests in Mathematics and Swedish (%). Results in grade 3 are used for primary schools, and results in grade 9 are used for secondary schools*.

b *Foreign background is defined as students being born abroad, or born in Sweden with both parents born abroad*.

### Focus-Group Interviews

An interview guide, shown in Table 3, containing questions related to the COM-B areas of Capability, Opportunity, and Motivation was used in focus-group interviews. The questions aimed to explore the participants' experiences and perceptions of their roles as an implementation team and the initial implementation of IBIS at their schools. The guide was used to inform the interviewer on the areas needed to be covered, and to allow further investigation in dissenting as well as dominant opinions. The focus groups were scheduled at the convenience of the participants, and were conducted at the schools. Brief introductions were given before each focus group, in order for the researchers to introduce themselves and their purpose for conducting the interview. The interviews, which lasted 30 to 60 min, were all audio-recorded and transcribed by a research assistant.

**Table 3:** Interview guide using COM – B areas as a guidance.

**COM – B area**	**Questions**	**Sub questions**
Motivation	Why did you choose to work with IBIS? What do you think IBIS can contribute with to your school? How do you experience the interest in IBIS throughout the school? Do you think the school staff has got the capability to work with IBIS? Do you find it important to work with IBIS? Why? What do you think is important to maintain the interest and motivation to work with IBIS?	How did you become part of the IBIS team? How many people think it is important, do you think?
Capability	What do you know about IBIS? Do you think that the school staff has sufficient knowledge and understanding of: - Preventive interventions? - Challenging behavior? - Creating secure relationships? - Creating clear expectations? - To act proactive?	What is IBIS for you? Tell us more about that knowledge/ those strategies
	Do you have strategies/ any model at school today to work with the areas mentioned? Do you have any previous experiences in implementing evidence-based programs at the school? What abilities and skills are needed in your team to continue with IBIS? Does everyone in the IBIS-team know what is expected of you in the implementation work?	
Opportunity	Tell us how you built up the IBIS team? How do you experience the division of roles and responsibilities in the IBIS team? How have you organized meetings with the various work teams? How is the communication with the IBIS instructors? What resources are needed from the school leaders, the IBIS instructors and the IBIS communal center to succeed with implementation?	How is the communication with the principal if he/ she is not part of the team? How is the communication between the team and all staff at the school? Via regular meetings? How often occur the meetings? How are the meetings structured? Do you meet the instructors often enough?

### Coding and Thematic Analysis

Data were analyzed using thematic analysis and informed by a guide for using the Theoretical Domains Framework of behavior change to investigate implementation problems (Atkins et al., 2017). In the first stage of analysis, the data were coded into Michie et al. (2014) three COM-B categories of Capability, Opportunity, and Motivation. To ensure the reliability of the coding scheme, the first author coded two of the transcripts and then the second and third authors coded them anonymously according to Miles et al. (2014). Based on discussion among the three authors, the coding scheme was further revised. In the second stage of analysis, data coded into the three COM-B categories were further coded into TDF categories. At this stage, continuous discussions were held between the first and third author. When possible, the authors also tentatively categorized the participants' statements as either barriers or facilitators, based on previous research on barriers and facilitators in school implementation. The reliability of the coding process was ensured through continuous discussions between the first and third author and reliability checks as they coded the transcripts on their own at first and then checked the correlation rates. When the coded results had high amount of correlation between each other they were put in a result sheet where the themes were presented together with the number of schools that had mentioned the theme. Excerpts used in the result presentation were selected based on how well they represented the categories and codes. When a quote represented the perception of all schools it was highlighted in the result sheet and mentioned in the result section. When a quote only represented the perceptions from a few participants this was mentioned as well in the result. When the authors were finished with the coding and the result table and excerpts, the first author read the transcripts once more to make sure no results were missing.

## Ethical Considerations

The study followed the Swedish Research Council's ethical guidelines. The participants were informed of the study's purpose and of their freedom to choose whether to participate. Participation in the study was confidential, and participants' names were changed to codes in the transcripts and analysis of the interviews.

## Results

The focus groups revealed different experiences of the initial implementation of SWPBIS within the nine studied teams. The study found significant/relevant themes of TDF areas of implementation within the three domains of Capability, Opportunity, and Motivation, according to implementation team members' perceptions. Table 4 presents the themes from the interview transcripts, along with the corresponding TDF and COM-B dimensions. The themes are also divided into potential enablers and barriers based on previous research and the participants' perceptions. As seen in Table 4, the themes are evenly distributed over the COM-B dimensions. Apart from *physical skills*, it was possible to distribute the data over all TDF areas. However, a great deal of the themes were coded under the Opportunity dimension of environmental context and resources, and no themes were found under the Capability dimension of physical skills. The results are further presented aligning within the COM-B and TDF dimensions, including interview excerpts.

**Table 4:** Themes categorized in COM-B areas and aligning within the Theoretical Domain Framework and potential enablers and barriers.

**Themes categorized in COM-B and the TDF**	**Enablers**	**Barriers**
**Capability (knowledge)**		
Knowledge of program content	Understanding the aim of the program	Insufficient knowledge of the program content
**Capability (cognitive and interpersonal skills, behavioral regulation)**		
Process or result orientation	Process orientation: focusing on the step-by-step process and having patience with the results	Result orientation: Being frustrated and doubtful when doing the right thing without seeing any results
**Opportunity (Environmental context and resources)**		
Manual adaptation	The manual as helpful and trustworthy because of the evidence base	The manual as not user-friendly and not trustworthy due to adaptation
Time	Sufficient time is allocated to implementation	Goals and time conflicts
Organizational prerequisites in coordinating implementation activities	Implementation is incorporated into meeting and responsibility structures	Reorganization and staff turnover
**Opportunity (social influences)**		
Team functioning	Team is perceived as well-functioning	Unclear roles and responsibilities within the team
School and implementation leadership	Clear leadership	Unclear leadership
**Motivation (goals, intentions, and beliefs about capabilities)**		
Match between program and staff beliefs	The aim of the program matches the perceived needs The content appeals to the staff and organization	Doubts as to the evidence and effect of the program after adaptation adjustments
**Motivation (beliefs about consequences, optimism)**		
Expectations	Plausible expectations regarding the initial implementation outcomes	Expectations are higher than the initial implementation outcomes
**Motivation (professional role and identity)**		
Program as a unifying factor	Program as common platform and unifying factor	
**Motivation (emotional and reinforcement)**		
Perseverance/Reinforcement	Reinforcement via results and progress markers	Delay in the implementation process and insufficient implementation of reporting systems

### Capability – Knowledge

One theme was found under the TDF's Knowledge, labeled *knowledge of program content*. Knowledge of programs with a theoretic base similar to that of IBIS was perceived as a helpful start in understanding the aim of the program. Previous experience of proactive programs was perceived as something that led to a better understanding of the IBIS program's aim and content. For example, participants who reported having worked with proactive behavior strategies and relationship-building perceived a clear link between IBIS and previous programs. Others pointed out that content in IBIS was in line with what the school was already doing:

“What IBIS would contribute is in line with what we think we're already doing at our school.” (School 6)

The lack of knowledge and understanding was also described as a factor affecting the ability to implement the program. Several schools also described insufficient knowledge about the program as something that had complicated the initial implementation. They expressed dissatisfaction with the start of the implementation process, reporting that they had not received enough information about the program's content. They expressed a need for knowledge of what to do when working with IBIS, and why they should do it:

“It would have been better if the implementation team had gotten more training in IBIS first, to be able to spread it in the whole school… for example, if we'd had a basic course and training for one term so that we'd feel safer in the way we're heading. As it is now, I think it's difficult to lead the staff because I don't really know where we're heading.” (School 3)

### Capability – Cognitive and Interpersonal Skills, Behavioral Regulation

One theme was found under the TDF's Cognitive and interpersonal skills and behavioral regulation, labeled *process or result orientation*. Participants at some schools mentioned a need to focus on a step-by-step process of implementation as an essential skill in successful implementation. Participants expressed it as “we're being too quick” (School 9), “we need to be patient” (School 1), or “we need to be calm and let it take some time” (School 4). Participants pointed out that it is more common for schools to start than to maintain the implementation process, and that these maintenance skills are essential in the process:

“We need to support each other so we can manage to hold on to the program. This is what many schools do wrong. They start with something and then get tired of it after a year and say it didn't work.” (School 4)

According to the participants it is essential to understand that changes in student behavior take time, and several of them mentioned a need for patience:

“You need to have patience, but you want to see small things in the students that show you that we're on the right track.” (School 3)

Some participants reported frustration and difficulty in conducting implementation activities with school personnel when there was not enough to work with or insufficient results. They described a common frustration at doing the right thing but not seeing any results in the students. This quote exemplifies this frustration as building relationship, one of the core components in IBIS, takes time and the results might show first after several weeks of relationship-building:

“When you've actually done the right thing but there's a frustration that you're not aware that it's going to take another 10 weeks to build up the relationship with the kids so you can have a peaceful classroom.” (School 8)

### Opportunity – Environmental Context and Resources

Three themes were found under the TDF's Environmental context and resources: *manual adaptation, time*, and *organizational prerequisites in coordinating implementation activities*.

#### Manual Adaptation

Many teams appreciated the manual's research background. Participants stated that the fact that IBIS is a translation of the evidence-based program PALS, deriving from the evidence-based framework SWPBIS, made them trust its content. At the same time as the staff appreciated that the manual was extensive, including the research base and the theories behind SWPBIS, they also commented that they wanted it to be more flexible, with possibilities to pick up specific strategies and delve deeper into them; they also wanted complementary resources, such as interactive materials and films:

“It would have been fantastic just to push a button and have it kind of pop up, like here you can find… find references and short videos or video links or something like that!” (School 2)

“More workshops,” “exercises,” “demonstrations of how it should work,” “what it should look like in the classroom, purely practically.” (School 6)

Some teams even offered examples of how they had used the manual more as a source of support in proceeding with the implementation in a way that better suited the school:

“Now we choose the reading assignments we have or the method we're about to use and what the scientific foundation is … it can be a bit heavy, but still it wasn't that extensive. We talked about having to go back later, read it, try to memorize it, and then go back when we need to re-evaluate.” (School 5)

The results show that the participants appreciated the evidence base in showing adherence to the manual, at the same time as they experienced being bound to following the manual, which they experienced as too slow, inflexible, and not totally fitting the needs and competencies in the schools:

“Give the instructors a little more space to use their competencies with support from the manual, instead of the instructor starting with the manual and then binding herself to do it in a certain way. And then instinctively feeling that we should actually move a little faster in this area, but the manual binds me to do it this way instead.” (School 5)

“…you wait a little to get going. And then it might not always [work]. Maybe as a leader I should have taken a few steps myself. Maybe I should have taken more initiative. But I've waited for the next step a little and then it gets to be a little ‘uhh…”' (School 2)

The quotes show that the teams are calling for adapting the intervention and altering the pace of implementation to suit the needs of the school.

#### Time

Time was mentioned in all the team discussions. The participants pointed out that time is scarce and that schools are often involved in several projects that do not always have the same goal, and that there is a need for conscious prioritization. Several teams pointed out that they allocate time to the implementation of IBIS during staff meetings and that there is a need for time for implementation; not long time periods for meetings but rather regular meetings, actively creating teachers' task lists:

“The advantage at our school is that we have chosen to reduce engagement in other tasks so we can focus on the right things, and then we're able to work with the implementation, so we don't have so many forces dragging us in other directions.” (School 5)

At other schools the teams discussed lack of time, due to goal conflicts when many different projects are run in the school at the same time. They pointed out that working with IBIS requires full engagement, and that this can be difficult to achieve when specific time is not allocated for the implementation:

“…it takes engagement, it takes time, and if we don't have the prerequisites to do that, we're afraid it won't be good; it'll be kind of stressed, we won't be able to, we won't have time for, we won't have the engagement that's needed.” (School 1)

#### Organizational Prerequisites in Coordinating Implementation Activities

The schools seemed to have different organizational prerequisites for implementing the program. Some had organizational structures in place that were seen as useful for the implementation activities. At these schools, teams reported that manual components were incorporated into school routines, and the participants saw this as important. According to participants, this is done using existing school routines such as staff meetings, classroom lessons, and the overall school schedule. For example, one team described using so-called “VIP time” for the IBIS project. Another team described that they incorporated IBIS into school meeting routines.

“As it looks right now, we've put IBIS on the agenda for all staff meetings. We've checked staff training days for the whole school year and put an asterisk for IBIS, and then every week we have an opening for IBIS, so that we have a kind of “homework” for us as professionals every week.” (School 5)

“We've scheduled time every week with the students, 40 min of IBIS time … where we already work actively with values, prosocial activities, we do great work … and have a well-functioning friend team… safety board … student board, and health board, and we've also organized free time…” (School 8)

At one school, the implementation was begun during a reorganization of leadership and staff. This school reported having difficulty coordinating the implementation activities:

“The situation has been that the working environment and the challenges at the school have generally resulted in our getting two new principals. This means that we need more time to build up the organization necessary for the implementation, with us and the new principals.” (School 1)

### Opportunity – Social Influences

Participants described *leadership* and *team functioning* as affecting the implementation. These two themes were categorized under the TDF's Social influences and the COM-B dimension of Opportunity.

#### Team Functioning

Participants emphasized the importance of *a well-functioning team* in the implementation process. Characteristics of a well-functioning team were described in terms of group composition, roles, and responsibilities, as well as group atmosphere. For *group composition*, it was pointed out as important to have a team constituting professionals who have a strong position and key posts at schools. One team said “it's natural to have a special needs educator on the team” (School 5), another that “all the parts of school organization should be represented on the team” (School 6), and yet another that “we're doers” and “it's a good atmosphere, a really good mix of formal and informal positions” (School 7). Clear responsibilities were seen as important in making the team work smoothly. This is exemplified in excerpts such as:

“We're really a good team with clear roles” and “we've tried to divide our roles and try to stick to the roles.” (School 9)

Some teams pointed out that there could also be an absence of clear responsibilities:

“So, I didn't understand what my role versus your role is. I thought it was unclear.” (School 1)

Group atmosphere was also discussed as important:

“We have a good atmosphere within the team. We come unbiased to team meetings, and if we don't understand we can say it.” (School 6)

“It's open discussions within our team.” (School 9)

#### School and Implementation Leadership

The teams pointed out that leadership on the IBIS teams and at the schools was important for the implementation. Several teams commented on the importance of having a leader “to turn to when you have questions” (School 5). Overall, the participants regarded it as essential to have well-functioning communication with school leaders. The participants referred to leadership both as the leadership they experienced from the instructors and school principals, and as a key factor for successful implementation.

### Motivation – Professional Role and Identity

Under the COM-B dimension of Motivation and the TDF's Professional role and identity, one theme, labeled *Program as a unifying factor*, was found in all interviewed teams. All participating groups agreed that implementing IBIS could offer a chance to gain a common platform and unify the school personnel in their way of understanding the students' needs and finding solutions to problems. For example, participants stated that their reason for joining the IBIS implementation was to unite all the staff in a common goal and to acquire tools to engage everyone. All participants agreed that there was a need in the schools, and a potential in IBIS, to find consensus around how to treat and understand students:

“.. this work is a way to find a common approach and consensus in different situations.” (School 4)

### Motivation – Goals, Intentions, and Beliefs About Capabilities

Under the TDF dimensions of Goals, Intentions, and Beliefs about capabilities, one theme was found, labeled *match between program and staff beliefs*, in which participants emphasized high motivation because of the match to school needs. According to many participants, one reason why the school had chosen to implement IBIS was that the program's aim matched the perceived needs at the school. They stated that all staff would likely agree about the need for a more peaceful, calm school environment, better staff-student relationships, and fewer conflicts. The quote below illustrates that participants saw IBIS as a chance to encourage study peace and student motivation:

“It's a great motivation regarding these kinds of problem situations that we'd like to address and that most of us want to work with.” (School 7)

Somewhat different needs in relation to the program were discussed on the different teams. All teams expressed a need to tackle children's disruptive behaviors, but already from the start had different approaches to this mission. At some schools, the entire school staff agreed about the need for proactive approaches to tackling disruptive behaviors through work with common values, in order to work with the antecedents rather than the consequences of students' behavior:

“The importance of classroom norms caught our eye and it felt really right … it doesn't focus on specific students but rather the whole class.” (School 5)

At other schools, seeing disruptive behavior as a challenge, the teams reported that the staff were skeptical to the proactive and preventive approaches in Tier 1 of SWPBIS:

“We have broad work directed at everyone, value-based work in different ways, but it might not reach those who are usually involved in real problematic situations; they need something else… then I think we have a group of around 20 or 10%, maybe, who think it's hard work to confront their own way of being; it's always that way. And they're not open to doing anything right now. But we can't wait for them either, so... ” (School 7)

### Motivation – Beliefs About Consequences and Optimism

Under the TDF's Beliefs about consequences and Optimism, one theme, labeled *expectations* – whereby participants referred to expectations both as being too high and as plausible – was found as something affecting motivation for the implementation. The ability to have plausible expectations was something that arose in most teams as an essential factor in the initial implementation. Two teams talked about the importance of showing the staff what is happening as an important factor in keeping their motivation up:

“That there's a common thread, that everyone can see that we're working with this and that it all sticks together.” (School 1)

Many participants reported that school staff had high expectations at the start but experienced a slow start with insufficient action or results:

“So the expectations were very high, I think. And you think that this will all start with a big bang and it'll be a big thing, because it's quite a big thing to invite all the staff to a lecture where you're invited to be involved and included, to understand what it's about, and then it's a bit of an anticlimax when it suddenly dropped” (School 6)

### Motivation – Emotional and Reinforcement

Participants pointed out the importance of seeing the results of their efforts and noticing that what they are doing leads to something. This theme was labeled *behavior reinforcement* and was categorized as the TDF's Emotional and Reinforcement and the COM-B's Motivation, as it concerns the staff's experience of progress or results as motivation to continue the new behaviors. Many participants underlined the importance of reinforcement via results or progress markers for continuing the implementation process. For example, they stated that the hands-on strategies in the manual had been very important as they allowed the staff to first try the strategies on their own and then with their students; this helped them see that the strategies worked and helped them, and they noticed the gains when using hem. The participants stated that acting and seeing immediate results was a self-reinforcing activity. One participant expressed this as follows:

“That you get to try right away. I think that's important. That everyone gets to be involved and feel like something happens and we see the results.” (School 4)

“At the same time, I think it's good for them to see some effects. To make them catch the wave.” (School 9)

A few teams also reported that not seeing results discouraged them from continuing to work with the program. According to them, school personnel expected that more things would happen as the school started working with IBIS but lost their belief in IBIS as they perceived the start as slow. One participant stated the importance of reinforcement as follows:

“The first steps we take need to lead to results in some sort of way… so that we don't end up thinking that this is no better than anything else.” (School 2)

### Differences Between the Different Schools

Table 2 gives us information about the schools and show that three schools (1, 3, 9) included secondary level while the other schools only included primary level. In line with previous research on difficulties implementing PBIS at secondary level the result showed that many barriers that can be related to staff buy in (Motivation) or result orientation (Capability) were mentioned by all the three secondary level schools. Further on the schools differed in percentage of parents with post-secondary education. School 5 (32%) and School 6 (88%) differed the most in this aspect even though no differences could be found in the school excerpts. School 5 also differed from the other schools in having the lowest percentage of students meeting the grade requirements and percentage of students with foreign background. Still the perceptions of the initial implementation did not differ from the others. School 5 had many quotes matching the TDFs aligning with the COM-B area Motivation as Goals, Intentions, and Beliefs, Capabilities as well as the COM-B area Opportunity. Differing from other schools, School 5 had many organizational prerequisites on place and a high match between program and staff beliefs. Further on School 2 differed from the other as they had a lower rates of students per teacher (11) and a lower percentage of licensed teachers (74%). This school described differences in staff knowledge both as a motivation to use the program as a *unifying factor* (Motivation – professional role and identity) and as a barrier in implementation (Capability - cognitive and interpersonal skills, behavioral regulation). The latter theme was not coded as a main theme in the analysis as it was only one school having this discussion.

## Discussion

The aim of the study was to explore school implementation teams' perceptions of barriers and enablers in the initial implementation of the SWPBIS at nine Swedish schools, in order to gain a greater understanding of factors that hinder or enable the initial implementation processes in schools. Before discussing the study's implications, it is important to point out its limitations. First, the participants' discussions of barriers or facilitators in the implementation may be attributed to the quality of the support they received during the implementation. Although, as evident in the description of the implementation in the Method section, the implementation closely followed the stages of implementation (Fixsen et al., 2011, 2013) and the PBIS recommendations (Horner et al., 2014) and N-PALS (Arnesen et al., 2003), it may have happened that the school professionals' discussions of barriers to implementation reflect the possible lack of support provided. Second, the results should be interpreted with caution, as no data on the actual quality of implementation in the nine schools, besides the instructors' reports, were available to the researchers. The absence of whole staff perception prior to team training and implementation also limits the ability to draw any conclusions as to the relationship between the training of the teams got and their ability to implement SWPBS with the staff. As the interviews were done in a very early stage of implementation this study can only explore the initial perceptions of the intervention, no conclusions can be drawn on fidelity or quality of implementation. Furthermore, the implementation teams' participation in the focus groups was likely more focused on the IBIS model. Less motivated practitioners may have had additional views on barriers and enablers. Focus groups also run the risk of being biased due to the desire to conform to social acceptability. Another limitation is that the participants in the interviews had no possibility to check the transcripts before the interpretation process started. There may also have been a risk of bias in the data interpretation, as the researchers might have had preconceived ideas about implementation in Swedish schools based on their prior knowledge and experience.

Despite the limitations of the study, its results make important contributions to the field of implementation of positive behavior interventions and support at schools. This study opens up for a new context (Swedish schools) and a new approach to understanding enablers and barriers in the initial implementation of SWPBS. Still, this is an interview study on a small number of participants. Further research, using a mix of quantitative and alternative qualitative methods, is needed to investigate and validate the results of this study.

Viewed through the lens of the model of behavior change (Michie et al., 2005, 2011; Cane et al., 2012), the professionals' views regarding barriers and enablers in three components were identified. Regarding Capability, the interviewed professionals viewed the knowledge and experience of similar evidence-based programs as well as interpersonal skills in implementation of programs as important. Concerning Opportunity, the interviews revealed environmental context such as time, the manual, and organizational prerequisites, but also social influences such as team functioning and leadership, as important factors. Finally, several facilitators and barriers were related to the Motivation domain. The professionals expressed that their decision to enroll in the PBIS implementation was based on seeing the program as a unifying factor, and it was important that the program aligned with their beliefs regarding addressing challenging behaviors. Furthermore, beliefs about consequences and emotional reinforcement were viewed as important in the initial implementation of the program.

Although PBIS has shown promising results in previous research, these results have shown to be dependent on the implementation (Bradshaw et al., 2008; Horner et al., 2009; Dix et al., 2012; Flannery et al., 2014; McIntosh et al., 2014; Sørlie et al., 2018). Initial implementation is one of the most critical stages in program implementation (Nese et al., 2016), and this study used an implementation model of behavior change, COM-B, to investigate enablers and barriers in the initial implementation of PBIS in Swedish schools.

### What Core Features Do the Implementation Teams Perceive as Hindering or Enabling in the Initial Implementation of the Swedish Version of SWPBS?

The results of this study corroborate those of previous studies, revealing the importance of organizational prerequisites in terms of leadership, accurate training, and sufficient time and resources for the successful implementation of SWPBS (Kincaid et al., 2007; Lohrmann et al., 2008; Chitiyo and Wheeler, 2009; Flannery et al., 2009; McIntosh et al., 2013; Horner et al., 2014; Pas et al., 2015; Tyre and Feuerborn, 2017; Goodman-Scott et al., 2018; Tyre et al., 2018), and proper planning of staff and resources and proper training (Kincaid et al., 2007; Lohrmann et al., 2008; Chitiyo and Wheeler, 2009; Bambara et al., 2012; McIntosh et al., 2013; Pas et al., 2015; Tyre and Feuerborn, 2017; George et al., 2018; Goodman-Scott et al., 2018; Tyre et al., 2018). Thus, these factors appear to be important in the initial implementation of PBIS at schools.

The importance of these factors has been acknowledged in previous research on the implementation of PBIS. The implementation of PBIS is guided through well-defined implementation stages, and before a school begins the implementation, they are asked to conduct an initial exploration of the school's readiness for the program and make time and organizational space for the implementation activities (Norwegian Center for Child Behavioral Development, 2017; Sugai and Horner, 2020). The results in this study show that some implementation teams reported having the necessary time and resources while others did not. Thus, despite similar support from the researchers, the enrolled schools varied in their perceptions of the implementation processes. Even though a key component of IBIS is preparing the organization for implementation, the results show that this can be a difficult task to achieve for some schools due to circumstances beyond their control, such as change of leadership or short notice of the project start. Competing agendas in the choice of evidence-based practice and the need to cater to diverse populations of students have been identified as challenges in the implementation of evidence-based practice at schools (Wandersman et al., 2008; Cook and Odom, 2013; Forman et al., 2013). Previous attempts to implement programs based on the PBIS framework in the Swedish context have resulted in unsuccessful implementation and low program fidelity and shown problems with process management and collaboration problems as considerable barriers (Ingemarson et al., 2014; Bodin et al., 2016). Therefore, schools implementing SWPBS in Sweden would benefit from prioritizing the recommendations concerning planning and preparation in the initial implementation (Sugai and Horner, 2020).

The initial implementation is often a turbulent time, as the program content and associated routines are new to all participants (Fixsen et al., 2011, 2013). A factor that could help during this turbulent time is the school staff's ability to plan the implementation process. The teams in this study had varying approaches in this regard. Some teams expressed a frustration at the process being too slow or unclear, while others described their strategies as a step-by-step process of implementation, being aware that implementation takes time and effort. Overall, the teams asked for more flexibility and adaptability to adjust the pace and the content to fit the staff and organizational needs. In addition, the teams also reported that knowledge and experience of previous programs, focusing on proactive behavior support, enabled them to see the links more clearly to IBIS, while a lack of knowledge was seen as a barrier. Teacher experience and skills have been identified as important for implementation (Kincaid et al., 2007; Chitiyo and Wheeler, 2009; Bambara et al., 2012; Lohrmann et al., 2013; Horner et al., 2014; Pas et al., 2015; George et al., 2018). As evidence-based practice is widespread in schools, there may be a need to consider schools' previous knowledge and experience of program implementation. Thus, schools with less experience or knowledge may require more support than those with knowledge and skills acquired through evidence-based programs. Furthermore, helping teams at an early stage embrace a more process-oriented approach to implementation rather than a result-orientated approach could be an important aspect to address in the initial implementation of evidence based practice in schools.

An important factor for behavior change that was focused on in this study is the motivation to implement the program (Michie et al., 2005, 2011; Cane et al., 2012). One essential factor in the installation and initial implementation of PBIS is securing sufficient staff buy-in (Chitiyo and Wheeler, 2009; Forman et al., 2009; Lohrmann et al., 2013; Tyre and Feuerborn, 2017), something that N-PALS promotes in its attempt to get at least 80% of staff to engage in the implementation (Norwegian Center for Child Behavioral Development, 2017). Given the importance of staff buy-in and a history of opposition to behavior-oriented programs in the Swedish school culture and lack of consensus on what sort of innovations the Swedish schools need (Ingemarson et al., 2014), it was interesting to explore what professionals at Swedish schools consider to be important motivational aspects in their decision to implement PBIS. This study revealed that a perceived alignment between the program and professionals' beliefs and needs, and the program being a unifying factor, were important in professionals' decisions to enroll in the implementation of PBIS. These aspects are in line with what Moore et al. (2013) define as philosophical fit – how the program content matches the school personnel's values and aims – and appear to be important to address in the implementation of PBIS in Swedish schools.

Another relevant theme was that teams highlighted the importance of having plausible expectations and emotional reinforcement. While some of the interviewed teams in this study expressed frustration at the process being too slow, others underlined the importance of reinforcement via results or progress markers in continuing the implementation process. These teams saw it as important to highlight small successes achieved while implementing the program. Given the complexity and challenges in the initial implementation of PBIS reported in previous studies (Nese et al., 2016), these factors may need to be explored and targeted in future studies on the initial implementation of PBIS.

Finally, the professionals in the study discussed using the manual as an important aspect of implementation. Some teams reported that they needed to have the possibility to use it in a flexible way, being able to pick up specific strategies and delve more deeply into them and not be restrained by the manual. This can be related to the discussion of the fit between the program and local conditions at specific schools. Previous research emphasizes the importance of the ability to adapt to the organizational needs and prerequisites in the initial implementation in schools (Fixsen et al., 2013). Although it may be in conflict with program fidelity, according to von Thiele Schwarz et al. (2019), adaptations in programs are vital in ensuring intervention-context fit and the value of a program to the recipients of it. The N-PALS offers schools opportunities to adapt, as long as the adaptation is within the framework of the model (Norwegian Center for Child Behavioral Development, 2017). The need to adapt the program to a school's local context and select evidence-based interventions with regard to a school's specific needs is emphasized in the PBIS framework (Sugai et al., 2009). The ability to adapt to school needs might be of extra importance in the Swedish school context as the school system allows for schools to be autonomous and differs widely in organization, ideological values, and teaching methods (Helgøy and Homme, 2007; SOU, 2020:28). Further on the importance of scientifically based methods has been highlighted in Swedish schools at the same time as the system of independent schools has grown stronger in the last decade (Adolfsson and Sundberg, 2018). Thus, the possibilities to adapt the IBIS implementation is an aspect that may need to be further stressed in the continued implementation of the program in Sweden.

### How Can the COM-B Model and the TDF Framework Enhance the Understanding of the Initial Implementation of SWPBS in a Swedish Context?

A second aim of this study was to explore the usefulness of the COM-B model and the TDF in enhancing the understanding and user-friendliness of the initial implementation of SWPBIS in a Swedish context. As implementation entails staff behavior change, a framework of theories based on psychological knowledge of behavior change offers a unique framework for exploring barriers to and enablers of implementation (Atkins et al., 2017); this study shows how it can be used to further understand the initial implementation of SWPBIS.

Using COM-B as a model and the TDF as a framework for analysis guided by Atkins et al. (2017), several relevant barriers and enablers in line with previous research were found, as discussed above. However, some identified determinants, such as process orientation, program as a unifying factor, and emotional reinforcement, give us new and further information on what can encourage staff buy-in and how teams adapt to insufficient circumstances. One of the reasons why these factors were found might be that a new framework, in the field of PBIS research, was used in the analysis. The framework has been widely used in explorative and quantitative studies eliciting the perceptions of professionals and has in this study shown its potential to explore implementation problems as initial implementation in schools and cultural adaptions of PBIS in a Swedish context. As mentioned above, adaptations are needed for successful implementation in schools. The TDF domains can help future studies find information about what factors have the best prerequisites for use in an adaptation process as it covers organizational as well as individual aspects of behavior change.

At the same time, the domains could also tend to limit the production of themes, as they might have been missed if they did not match the COM-B questions or the TDF analysis. Using a framework to guide thematic analysis always might come with risks of self-confirmation bias. Although the themes were evenly distributed over the domains, the domain of physical skills was not used. This might reflect the fact that the model was not specifically designed to fit school behavior change. At the same time, the model of behavior change shows how different factors need to collaborate with each other for behavior change to occur (Francis et al., 2012; French et al., 2012), and can compensate for each other in the process of initial implementation.

## Conclusions

This study used the Theoretical Domains Framework (TDF) and the COM-B model to look at what factors school professionals view as important for behavior change, related to Capability, Opportunity, and Motivation. While several factors with regard to Opportunity have been investigated in previous research (e.g., Horner et al., 2014; Tyre and Feuerborn, 2017; Goodman-Scott et al., 2018; Tyre et al., 2018), this study contributes important aspects that may be targeted in initial implementation with a focus on capability and motivation for staff behavior change. These aspects can be addressed to compensate for a loss of opportunities and the contextual challenges many schools face in an initial implementation process (Fixsen et al., 2013; Nese et al., 2016).

The results implicate further development of the implementation process of IBIS in Swedish schools. These recommendations could be relevant in the implementation of SWPBS in other countries, but need to be further researched. Even though the TDF and the COM-B were originally developed to identify influences on health professional clinical behavior change (Francis et al., 2012), the model has recently been used in a school context (e.g., Clarke et al., 2015; Bayley et al., 2017; Martin and Murtagh, 2017; Hatfield et al., 2019). This study is the first to use the TDF to explore the initial implementation of SWPBIS, and the usefulness in finding new relevant information indicates the need for further exploration with the TDF as guidance and the COM-B as a model for understanding. Future research could especially benefit from using the perspectives of Capability and Motivation in implementation in schools. This study reveals the need for adaptation in the initial implementation of SWPBIS. Even though the adaptability of SWPBIS is well-developed, further research is needed to determine what factors can compensate for challenges that can be difficult to change in the initial phase, and how these factors correlate to program fidelity and sustainability.

## Data Availability Statement

The raw data supporting the conclusions of this article will be made available by the authors, without undue reservation.

## Ethics Statement

Ethical review and approval was not required for the study on human participants in accordance with the local legislation and institutional requirements. Written informed consent for participation was not required for this study in accordance with the national legislation and the institutional requirements.

## Author Contributions

KN was responsible for study design, interview preparation, data analysis, research questions, author cooperation, and overall manuscript work. MK was responsible for data collection, description of main parts of the method, support in data analysis and the quality of research. NK was responsible for the quality of research, analysis and manuscript. Support in all parts of the process. TO was responsible for overall quality of research, background description and manuscript as a whole in the field of implementation and SWPBS research. All authors contributed to the article and approved the submitted version.

## Conflict of Interest

The authors declare that the research was conducted in the absence of any commercial or financial relationships that could be construed as a potential conflict of interest.

## Nomenclature

### Resource Identification Initiative

To take part in the Resource Identification Initiative, please use the corresponding catalog number and RRID in your current manuscript. For more information about the project and for steps on how to search for an RRID, please click here.

### Life Science Identifiers

Life Science Identifiers (LSIDs) for ZOOBANK registered names or nomenclatural acts should be listed in the manuscript before the keywords with the following format: urn:lsid:<Authority>:<Namespace>:<ObjectID>[:<Version>] For more information on LSIDs please see Inclusion of Zoological Nomenclature section of the guidelines.
